# Creating an mHealth App for Colorectal Cancer Screening: User-Centered Design Approach

**DOI:** 10.2196/12700

**Published:** 2019-05-08

**Authors:** Lauren Griffin, Donghee Lee, Alyssa Jaisle, Peter Carek, Thomas George, Eric Laber, Benjamin Lok, François Modave, Electra Paskett, Janice Krieger

**Affiliations:** 1 STEM Translational Communication Center University of Florida Gainesville, FL United States; 2 Department of Community Health and Family Medicine University of Florida Gainesville, FL United States; 3 Division of Oncology College of Medicine University of Florida Gainesville, FL United States; 4 Statistics Department North Carolina State University Raleigh, NC United States; 5 Department of Computer and Information Science and Engineering University of Florida Gainesville, FL United States; 6 Center for Health Outcomes and Informatics Research Loyola University Chicago Chicago, IL United States; 7 Division of Cancer Prevention and Control Department of Internal Medicine The Ohio State University Columbus, OH United States

**Keywords:** communication, cell phone, mobile phone, culturally appropriate technology, interdisciplinary research, colon cancer, cancer screening

## Abstract

**Background:**

Patients are increasingly using mobile health (mHealth) apps to monitor their health and educate themselves about medical issues. Despite the increasing popularity of such apps, poor design and usability often lead to suboptimal continued use of these apps and subsequently to poor adherence to the behavior changes at which they are aimed. One solution to these design problems is for app developers to use user-centered design (UCD) principles to consider the context and needs of users during the development process.

**Objective:**

This study aimed to present a case study on the design and development process for an mHealth app that uses virtual human technology (VHT) to encourage colorectal cancer (CRC) screening among patients aged 50 years and above.

**Methods:**

We have first provided an overview of the project and discussed its utilization of VHT. We have then reviewed UCD principles and how they can be incorporated into the development of health apps. We have described how we used UCD processes during the app’s development. We have then discussed the unique roles played by communication researchers, computer scientists, clinicians, and community participants in creating an mHealth app that is credible, usable, effective, and accessible to its target audience.

**Results:**

The principles of UCD were woven throughout the project development, with researchers collecting feedback from patients and providers at all stages and using that feedback to improve the credibility, usability, effectiveness, and accessibility of the mHealth app. The app was designed in an iterative process, which encouraged feedback and improvement of the app and allowed teams from different fields to revisit topics and troubleshoot problems.

**Conclusions:**

Implementing a UCD process contributed to the development of an app, which not only reflected cross-disciplinary expertise but also the needs, wants, and concerns of patients.

## Introduction

### Background

As technology improves, patients are increasingly using mobile apps to monitor their health and access medical information [1]. More mobile health (mHealth) apps are entering the market every year. However, poor development may diminish the usefulness of apps to patients [2]. Many mHealth apps are downloaded by patients but rarely used [3]. As such, it is recommended that teams developing mHealth apps use processes that consider the context and needs of users [4].

Over the past decade, access to the internet and smartphone ownership have increased to the point that virtually everyone in the United States has access to digital information. Furthermore, approximately three-quarters of Americans (including two-thirds of rural residents) have regular internet access [5]. Almost all Americans own a smartphone [6]. There is significant evidence that the penetration of the internet and mobile technologies could completely transform the way health care is delivered. It has the potential to effectively and efficiently deliver health behavior interventions with unsurpassed scalability [7-11]. Nonetheless, an expanding body of literature suggests that digital interventions lack the evidence-based standards required for apps to be usable in a health care setting or recommended for home use by health care providers [12-17]. The suggested reasons for lack of quality are lack of physicians’ and patients’ involvement in the development of such digital interventions. Although some recent research initiates strategies to involve stakeholders, this is not widespread yet [18].

This study presents a case study on the design and development process for an mHealth app that uses virtual human technology (VHT) to encourage colorectal cancer (CRC) screening among patients aged 50 years and above. Using participant observation, semistructured interviews, and document analysis, we have described the process by which a multidisciplinary team developed the app. The outcome was an mHealth app that reflects best practices across the medical, communication science, and computer science fields.

We have first provided an overview of the CRC screening project and discussed how it incorporates VHT. We have then reviewed the principles of user-centered design (UCD) and why UCD is useful for developing mHealth apps. We have described how the UCD process played out during the app’s development, with a particular focus on how each set of researchers contributed to the overall design during each phase. In doing so, we expounded upon the unique roles played by communication scientists, computer scientists, clinicians, and community participants in creating an mHealth app that is credible, usable, effective, and accessible to its target audience. Our goal was to offer insights into the development process for other teams working on mHealth technology.

### The Importance of Colorectal Cancer Screening

Among American men and women, CRC is the second leading cause of cancer death [19]. Racial and ethnic minorities are disproportionately impacted by CRC, with elevated incidences and mortality [20]. Although regular screening increases CRC detection and survival [21,22], minority patients face barriers such as time and monetary constraints and aversion to traditional screening procedures such as colonoscopies [23,24]. Similarly, rural patients are also disproportionately impacted by CRC morbidity and mortality [25]. Rural patients are less likely to understand the importance of screening and perceive cost as a barrier [26,27].

Fecal immunochemical testing (FIT) is a CRC screening procedure that may minimize the perceived barriers. Patients collect a stool sample at home and send it to a laboratory to test for microscopic blood that may indicate a tumor or colonic premalignant polyp. For patients at average risk, annual FIT is as effective as colonoscopy in detecting CRC [28,29]. As patients complete the test at home, FIT reduces barriers such as time, cost, and discomfort with colonoscopy. FIT is effective at increasing screening compliance for racial and ethnic minorities and rural populations [30].

### Virtual Human Technology

VHT consists of computer-generated animated characters that can be used to communicate with people using speech or text [31]. VHT is increasingly common in health care. The technology has been used in studies on mental health care [32,33], assessing pain treatment [34-36], and patient and provider communication [37]. VHT has been used to increase patient satisfaction [37], improve the understanding of cancer risks [38], and give hospital discharge instructions [39]. The term *virtual human technology* is used specifically to describe three-dimensional human characters. This is different from an embodied conversational agent (ECA), which can be any anthropomorphic character, including a human. In other words, VHT is more specific than an ECA. VHT is also different from a chatbot, which is more general and includes all systems that can converse with users.

VHT may be useful for increasing CRC screening compliance for several reasons. Patients may feel more at ease discussing sensitive information because of VHT’s sense of anonymity [40]. It may encourage patient disclosure [31], and it can also be used to provide tailored health information for patients, increasing perceptions of relevancy [41]. Similarly, demographic discordance between minority patients and providers is associated with worse medical outcomes [42-44]. VHT can match patients with demographically concordant virtual providers.

### User-Centered Design

The design of an mHealth app impacts its use and effectiveness. As Schnall et al point out, many apps fail because they are not designed to meet the requirements of the people who are actually using them [4]. Such apps are unlikely to be used by patients [3]. Developing apps using a UCD process may address these shortcomings [3,4,45].

UCD is a multidisciplinary, iterative design process that involves actively seeking out and incorporating the feedback of users to ensure that tools are developed with a full understanding of their needs and requirements [46]. In UCD, social scientists act as translators between users and designers, using their research skills to collect and interpret data about users and their needs [47].

The first phase in UCD is *needs investigation*. The goal of *needs investigation* is to identify potential users and learn about their specific needs for an mHealth app [3]. Many methods can be used in *needs investigation*, including cultural probes [48], interviews [49], and focus groups [3,4]. The second phase is *prototype development.* During *prototype development*, a trial version of the app is developed and tested, incorporating user feedback at multiple points [50]. The third phase is *evaluation*. During *evaluation*, researchers watch users test and evaluate the near-final app before rolling it out to larger audiences. Observing users can show researchers specifically how participants use the app and what problems they may experience [47]. These tests show researchers how the app functions when used by the type of people who will eventually use it on their own.

Although conceptually clear, in practice these phases are rarely clear-cut. As UCD is iterative, phases may blend together as researchers refine the app, troubleshoot problems, and seek additional feedback from users. This iterative process keeps the focus of development on users and ensures that the final product meets their needs [50].

## Methods

First, we collected notes, meeting agendas, and other written documentation produced during the early stages of development. Second, the study’s lead author engaged in participant observation of the development process, working as a postdoctoral researcher on the project while taking notes and working with the team on the app. Finally, the lead author interviewed 6 members of the development team about their role in the development process. The interviews were evaluative, approximately half an hour each, and transcribed for analysis.

A multiyear grant from the National Institutes of Health funded the development of the app. The design project is based at the University of Florida (UF), and the app will be a part of a clinical trial conducted at the UF Health Network, including Shands Hospital, launched in 2018. Furthermore, 3 core teams—clinical medicine, communication science, and computer science—contributed to the development of the app.

The app features an interaction with Agent Leveraging Empathy for eXams (ALEX) , a virtual human health care provider who educates patients about CRC screening and the benefits of FIT. During the clinical trial, we screened out patients who were at high risk of CRC (patients whose providers request more frequent colonoscopies or who have had colon cancer in the past) and those who were already within guidelines. Patients who are eligible for FIT see a series of tailored messages about CRC and its severity, their susceptibility to the disease, and how FIT can help them comply with screening guidelines. After visiting with ALEX, the app delivers an electronic message to patients giving them the option to request FIT from their primary care provider (PCP).

The app integrates into the UF Health Network and is delivered to patients directly through MyUFHealth (formerly known as MyChart), a Web-based medical portal. MyUFHealth lets patients securely access medical records, view laboratory results, and communicate with their PCP [51]. There are several advantages to integrating with MyUFHealth. First, using MyUFHealth to disseminate the app allows us to select patients with specific medical characteristics (ie, outside guidelines and average risk) for participation in the trial. Second, integrating with MyUFHealth lets us customize ALEX based on the demographic information in the patient’s file. Finally, using MyUFHealth allows patients to quickly and securely request FIT from their PCP.

## Results

### Overview

The next section discusses how the UCD phases (needs investigation, prototype development, and evaluation) played out in the development of the CRC screening app. It focuses on the contributions of the communication science, computer science, and clinical teams to the credibility of the app, its usability, effectiveness, and accessibility. As UCD is iterative, many development processes happened simultaneously. The team often circled back to questions and concerns raised earlier in the process. Similarly, we sought and incorporated feedback from participants at multiple points in the development. As such, this section should be seen as a streamlined overview of the development process, which by necessity simplifies some elements.

### Development Structure

We structured the development process around regular meetings between the 3 teams. The communication science team held weekly core meetings to coordinate development progress and integration into the larger university health system. The communication science and computer science teams met twice monthly to work on the hardware and software design of the app, with the communication science team providing feedback from potential users. The communication science and computer science teams also met with information technology (IT) representatives from UF Health as needed. We held these meetings in-person or online using a virtual meeting service. All 3 teams—communication science, computer science, and clinical—attended blended virtual and in-person meetings monthly and in-person meetings biannually.

This structure ensured that all teams understood how the app and clinical trial were evolving, even if they were not directly involved in a given branch of the work. It created flexibility for individual teams to meet as frequently as needed to accomplish their goals. Thus, individual teams could troubleshoot problems in a small-group setting and larger issues could receive input from all teams. We gained valuable feedback representing different disciplinary perspectives.

### Phase 1: Investigating Needs

As the project began, teams addressed 3 foundational app components (1) the content of the app, (2) the integration between UF Health and the app, and (3) the app’s software and user interface. During this phase, we developed the app conceptually, tested acceptability to our target audience, and began creating the software.

#### Communication Science and Clinical Teams

The communication science team and clinical team began by identifying the medical content necessary for the app, specifically what it would need to convey to patients. The clinical team identified, through their experience with patients, common barriers to screening, including cost, time, and feelings of embarrassment caused by collecting a fecal sample. They paid specific attention to barriers that were common among minority and rural patients. To understand how clinicians address these barriers, the communication science team video-recorded a simulated conversation about CRC screening between a patient and clinician. A member of the clinical team played the role of the clinician and a member of the communication science team played the patient. The clinician described in lay terms the risks of CRC, the benefits of screening, and the biological changes that occur in older people, which raise the risk of CRC. This conversation formed the medical basis of script between the virtual human health care provider and the patient.

We also discussed the needs of clinicians and health care staff through over 50 interactions with the medical staff, including family medicine physicians, colorectal surgeons, health care administrators, patient navigators, and other players in the biomedical field. We asked questions about their processes and workflow when interacting with patients, incentives at the provider and practice levels for screening patients, and structural challenges in getting patients screened.

Through these interviews, we learned that physicians would likely welcome a tool to help them communicate about CRC with their patients. PCPs often have multiple topics to discuss with patients and limited time in which to do so. Providing patients with information about CRC before their appointment provides shared background for a conversation. Similarly, the amount of new information patients receive during an appointment can be overwhelming and stressful for patients, particularly those with lower health literacy. Providing some information beforehand reduces the amount of new information patients must absorb.

However, routine and regulation tend to govern medical environments. This means that physicians are unlikely to accept mHealth apps unless they fit into the regular workflow. mHealth interventions also cannot create extra work or take time away from patient care. These considerations informed the app’s development. They are particularly important for the long-term dissemination of the app, as physicians and medical practices are a key channel for widespread distribution and adoption of the app by patients.

#### Computer Science Team

The computer science team began development of the virtual human health care provider. ALEX was created using Adobe Fuse, a design program, and Virtual People Factory, an interpersonal simulation system [52]. The computer science team created different versions of ALEX for focus group testing, designing a total of 8 characters varying along 3 dimensions: age (younger vs older), race (black vs white), and gender (man vs woman). They also had versions of the character in different attires, namely scrubs or business-casual office wear.

The computer science team began discussion of the hardware and software requirements of the app. With the larger team, they started the process of narrowing down which devices, browsers, and operating systems the app would support. As the app’s target audience is older adults (aged 50 years and older), they also brought up questions of accessibility. This included the need for subtitles and clear audio to accommodate visual and hearing impairments. Similarly, the app interface needed to be understandable for people with limited smartphone experience. These conversations continued throughout the development.

#### Community Involvement

The communication science team conducted 8 focus groups (n=36) with potential users from January to May 2017. Participants were aged older than 50 years, and the team held groups broken down by race and gender with black men, white men, black women, and white women. They recorded, transcribed, and analyzed the focus group data qualitatively. This first round of focus groups provided the team with valuable information about the preferences, needs, and opinions of potential users before prototype development.

Discussion centered around 4 areas: health information seeking (*What features make health information trustworthy?*), initial thoughts on the virtual human (*Would you be comfortable talking to a virtual human about your health?*), CRC knowledge (*What words or feelings come to mind when you think about CRCs?*), and attitudes toward FIT (*What are your initial reactions to the FIT kit?*). During the discussion, moderators showed participants still photos of different versions of the virtual human health care provider. The most important finding was that participants were open to discussing their health with a virtual human health care provider, providing an essential rationale for proceeding with the app development.

Overall, Phase 1 provided information on patient and clinician user requirements for the app. It established, through community involvement, the general acceptability of using a virtual human health care provider to encourage CRC screening. It also generated insights into the technical requirements of the app and potential accessibility challenges.

### Phase 2: Prototype Development

#### Computer Science Team

The computer science team had 2 main tasks during Phase 2: launching a working prototype of the app for user testing and planning the app’s integration with MyUFHealth. Developing the prototype required multiple steps including the animation of the virtual human health care provider, coding the internal logic of the app (including options for randomization for the clinical trial), and designing the user interface. The computer science team and the communication science team met biweekly to discuss progress and address potential problems, creating an iterative workflow.

For instance, syncing voice actors’ recordings of the script with the mouth movements of the virtual human health care provider required multiple iterations to reach an acceptable level. The communication science team originally asked colleagues in their college to serve as voice actors for a prototype ALEX. However, the varied speed and diction of nonprofessional voice recordings made it difficult for the computer science team to accurately sync the audio recordings with the lips of the virtual characters. To address this problem, the communication science team contracted professional voice actors to record the script. Paid voice actors recorded the scripts using professional equipment, which resulted in higher sound quality and greater syncing accuracy. The professional actors were also able to split audio files into segments to ease the process of syncing with the animation.

The computer science team began planning the app’s integration with MyUFHealth. As MyUFHealth is an existing platform with its own constraints, the team was originally unsure whether it would be able to house the app entirely or whether it would be necessary to host portions of the intervention on an external server. Using an external site would allow for easier tracking of users but raised security concerns. Particularly problematic was the need to import demographic information—considered Protected Health Information (PHI)—into the app to customize the virtual human health care provider. Finally, it was decided that the app would be housed on its own secure server and users sent customized links with encrypted identification codes that allow us to track their movements and responses as they worked through the app.

#### Clinical Team

During Phase 2, the clinical team gathered information about programs ongoing in the UF Health Network to encourage CRC screening. They sought to understand what clinicians were currently doing to increase CRC screening so as to avoid designing an intervention that duplicates ongoing work. This is important both from a messaging perspective—ensuring that patients are not receiving competing messages—as well as from an experimental perspective. In evaluating the effectiveness of the app during the clinical trial, it is important to understand and avoid confounding influences to the greatest extent possible.

The clinical team also collected information about screening rates at the various clinic locations and within the different departments at UF Health. This information allows us to evaluate the effectiveness of the app by comparing past screening rates with screening rates during the clinical trial. It also helps us account for influences such as seasonal variation in screening rates.

#### Community Involvement

The communication science team conducted 13 focus groups (n=73) from November 2017 through August 2018. All participants were aged between 50 and 73 years. Owing to changes in the recruitment process, we separated some focus groups out by race and gender and others by gender only. Participants first filled out a questionnaire gauging their perceptions of CRC risk and screening. They then tested the prototype app on a Samsung Galaxy S7 smartphone provided to them by the moderators. After engaging with the app, participants filled out a second questionnaire examining their opinion of the app’s technical aspects, the virtual human health care provider itself, and the CRC content. We recorded the focus groups and transcribed them for analysis.

The communication science team also held 38 think-aloud interviews during this timeframe, again using participants between the ages of 50 and 73 years. During think-aloud interviews, participants were asked to describe their thoughts and mental processes while using the app in real time [53]. The stream-of-consciousness data collected through think-aloud interviews let researchers see how participants are interacting with a tool, such as an mHealth app, in real time to better understand points of confusion and initial reactions.

Participants felt generally favorable toward the concept and script, with several indicating that they intended to ask their own PCP about FIT as a result of the experience. This provided preliminary evidence of the app’s potential acceptability and effectiveness. However, participants were critical of the virtual human health care provider’s appearance, indicating that the lack of a lab coat or medical name badge reduced the character’s credibility. They also expressed concern about the look and movement of the virtual human health care provider. Many found the virtual human health care provider *creepy* and *unsettling*, with several saying that they averted their eyes from the character and listened to the voice instead of engaging visually.

In February 2018, we held a meeting of our Community Advisory Board, a group of patients, advocates, and professionals in the medical field. At the meeting, we sought feedback from the Community Advisory Board on the prototype version of the app and script. As with the focus groups, the Community Advisory Board members felt that the look and movement of the virtual human health care provider was unrealistic and distracting. They also gave feedback on the script’s accessibility to those with lower literacy and/or health literacy and suggested areas within the script that needed to be expanded.

#### Communication Science Team

The communication science team incorporated the medical information collected during Phase 1 into a conversational script for the virtual human health care provider. They structured the conversation with ALEX around empirically-based constructs regarding CRC communication best practices. The original script identified 12 tailoring dimensions such as perceived susceptibility [54], perceived severity [55], perceived benefits [56], perceived barriers [23], self-efficacy [57], response efficacy [28], comparative risk feedback [58], risk probability [59], message source [60], narrative persuasion [61], demographic matching [62], and message framing [63]. Evidence suggests that these constructs can increase knowledge of cancer risks and screening and encourage behavioral change.

The team refined the script through input from multiple writers and readers, as well as the full app team and Community Advisory Board members. This led to significant changes, improving the script’s flow and understandability. The team also collapsed some constructs together for analytical purposes. Although the experimental design can accommodate multiple variables, analysis is complicated by each additional construct. The final message constructs are message source, susceptibility, severity, risk probability, response efficacy, benefits, barriers, narrative persuasion, and self-efficacy.

### Phase 3: Evaluation

#### Communication Science Team and Community Involvement

In Phase 3, the communication science team adapted the script and messaging to reflect community preferences gleaned from Phase 2. They clarified the constructs within the script for ease of analysis in the clinical trial and sent the script to an expert at the American Cancer Society to read for clarify, accuracy, and comprehensiveness. These comments, as well as additional feedback from the clinical team, were used to finalize the script.

The communication science team also tested the near-final app with community members by conducting additional think-aloud interviews between September 2018 and January 2019. We held additional 7 focus groups and 15 think-aloud interviews. The total number of focus groups throughout the process was 28 (n=154), and the total number of think-aloud interviews was 53.

The think-aloud interviews initially revealed that significant problems remained with the appearance of the virtual human health care provider, particularly the black female version. To address these concerns, the computer science team created alternative versions of the black female character for testing by the communication science team with subsequent think-aloud and focus group participants. At this point, the development of the app became more intensively iterative, with the communication science team providing rapid feedback to the computer science team on changes that needed to be made to the app to achieve minimal acceptability from participants.

#### Computer Science Team

The computer science team refined the app during the evaluation phase, making changes as a result of community feedback, in particular, the results of the think-aloud interviews and focus groups. This involved discussions with the computer science team about potential changes in the graphic approach to the virtual human health care provider’s appearance, moving from a more photorealistic look to one that was more stylized. The idea was that by going to a more stylized—but not cartoonish—look, participants would not be primed for photorealism and then put off by the limitations of the animation software and rendering process. Ultimately, the computer science team adapted models in Adobe Fuse to create a look that was somewhat stylized but also recognizable to viewers.

They also worked to integrate the app with MyUFHealth, ensuring that it was possible to demographically customize the virtual human health care provider for patients as per the study protocol. They paid particular attention to the need to track patients within MyUFHealth, as well as within the app itself, and the subsequent questionnaire (hosted on Qualtrics) and the need to link up these datasets for later analysis. They accomplished this through the aforementioned customized URLs and deidentification system. Using UCD principles helped ensure that the mHealth app we created was acceptable to patients along 4 major dimensions of user needs: credibility, usability, effectiveness, and accessibility.

## Discussion

### Principal Findings

By describing the creation of an mHealth app using UCD principles, we are able to better understand both the iterative nature of development when incorporating user feedback as well as the unique contributions of researchers across disciplines. Communication scientists, computer scientists, clinicians, and community participants all played specific and interrelated roles in ensuring that the final product was credible, usable, effective, and accessible for patients. We now summarize the specific components of these criteria and the contributions of each team in meeting them.

### Credibility (Clinical, Communication Science, Computer Science, and Community Involvement)

Credibility had 3 main components: (1) accurate medical information, (2) association with the UF Health Network, and (3) a professional look and feel to the app design. Community members were ultimately the arbiters of what app features were and were not credible, as interpreted by the communication science team.

First, the communication science team worked with the clinical team during Phases 1 and 2 to create accurate content that reflects best clinical practices. This is in line with recommendations that health interventions be designed with input from subject matter experts [64]. Indeed, focus group participants in Phase 2 raised questions about the app’s information source, with some explicitly asking whether UF Health was involved in development. Participants expressed skepticism about Web-based medical information, noting that such information is often misleading and inaccurate. However, they generally trusted the UF Health Network to provide them with credible information. Associating the app specifically with UF Health—a trusted medical provider—increased its credibility.

Second, the association between UF Health and trusted medical information was so strong that it carried over into participants’ preferences for the look of the virtual human health care provider. The prototype app tested in Phase 2 had ALEX in a business-casual outfit, and there was no visible association with UF Health. Patients described this look as unprofessional and said that putting the virtual human health care provider in a lab coat would increase credibility. The computer science team made these changes for the think-aloud interviews and focus groups in Phase 3.

Third, participants said an app needed to have a professional look and feel to be seen as credible. Participants in Phase 2 focus groups and early Phase 3 think-aloud interviews expressed discomfort with the look and animation of the virtual human health care provider. A key theme was that participants wanted the app to look like it was made by professional graphic designers to set it apart from other untrustworthy Web-based content. In other words, participants associated professional design and animation with medical credibility. Thus, even though clinical experts provided and vetted the app’s content, it took the skills of the computer science team to make that expertise visible to participants.

### Usability (Communication Science, Computer Science, and Community)

Usability had 2 main components: (1) intuitive app design and integration and (2) easily understood dialogue. As with credibility, community involvement helped operationalize these concepts in a way that reflected best practices from an academic perspective as well as from the perspective of the users themselves.

First, usability requires that the app design and interface be intuitive for both patients as well as clinicians and health workers. For patients, this meant that the app use and navigation needed to be self-explanatory even without instruction. Community feedback suggested a number of changes, which we incorporated into the app. For instance, the original working prototype had both a chat log and subtitles, which were seen as redundant. Similarly, although the app had a pause button, tapping the screen did not pause or play the interaction, which confused participants. Both these issues were corrected in the final version of the app.

For clinicians and health care workers, the app needed to intuitively fit into the clinical workflow to be usable, particularly with regard to requesting FIT. In designing this feature, the computer science team interfaced with UF Health to ensure that the appropriate medical professionals received the request through the appropriate channels, integrating with MyUFHealth. UF Health IT representatives indicated that clinical workers were accustomed to receiving information and requests from patients through the system. Using MyUFHealth, therefore, increased the usability of the app from the perspective of these employees.

Second, usability required that the app have understandable dialogue. This was a task taken up by the communication science team in translating the medical information from the clinical team into a coherent conversational script for ALEX. Multiple iterations of the script helped smooth out the sticking points in the dialogue, and feedback from a variety of readers increased cultural competency and eliminated jargon. Feedback from focus groups and think-aloud interviews suggests that these processes were largely successful—most participants felt that the app presented the information in an approachable and understandable way.

### Effectiveness (Communication Science, Clinical, and Community Involvement)

Effectiveness had 2 main components: (1) increasing knowledge of CRC and screening and (2) changing behaviors. Preliminary results from focus groups suggest that the app meets these aims.

First, in designing the script for the virtual human health care provider, the communication science team sought feedback from the clinical team and community to establish what participants were likely to know about CRC and screening. This hands-on input supplemented the information in the health communication literature on knowledge of CRC. It helped strike a balance between providing too much information (overwhelming or boring patients) and providing too little (leaving patients with more questions than answers). For instance, some participants in the Phase 1 focus group did not know what CRC was, incorrectly conflating it with prostate cancer and assuming that only people with prostates need to be screened. To remedy this shortcoming, the communication science team revised the script to describe CRC as *colon cancer* or *cancer of the intestine*.

Preliminary feedback from the focus groups indicates that the app is effective at increasing knowledge of FIT testing and its appropriateness for CRC screening. Many participants did not know about FIT testing before the discussion and were unaware that there were alternatives to colonoscopy. Indeed, many expressed surprise that there was such an easy option available for screening. Other participants were unaware of the specific risks of CRC before engaging with the app.

Second, the communication science team drew on information from the health communication literature and the clinical team’s expertise to write a script likely to change screening behaviors. For instance, both the literature and the clinical team stressed addressing barriers to screening, such as embarrassment about collecting a stool sample. To help lower these barriers and produce behavioral change, ALEX assures patients that they can complete the test in the privacy of their own home. This is important because messages that increase a person’s self-efficacy—or how much they believe they can influence an outcome—are effective at changing behaviors. People are more likely to take action if they believe it is effective in reducing a threat.

Although we will not have quantitative data about the app’s ability to produce behavioral changes until the end of the clinical trial, evidence from the focus groups suggests an increased desire to screen using FIT. Several participants asked how they could get FIT. Others explicitly stated a desire to use FIT, now that they knew it was effective. This suggests that the app will be effective at changing CRC screening behaviors.

### Accessibility (Computer Science and Community)

Creating an app that is accessible to the target audience relied on 3 main considerations: (1) using the correct technology to reach the audience, (2) ensuring that the app is easy to find, and (3) making the app accessible to audiences with different abilities.

First, the computer science team balanced the need to reach a wide audience with the developmental challenges of creating an app supported by different devices, operating systems, and browsers. Community participants in the Phase 2 focus groups illustrated this need. Participants typically accessed MyUFHealth from their desktop computers rather than their mobile phones. Many participants use MyUFHealth infrequently, increasing the likelihood of forgetting their username and password. Resetting the password on mobile devices is clunky, so participants defaulted to checking MyUFHealth from their desktop or laptop computers. Although we originally conceptualized the app as running mainly on mobile phones, the computer science team created a desktop version that increased the overall availability of the app for the target audience.

Second, the computer science team improved accessibility by integrating the app into the UF Health Network and MyUFHealth. Focus group participants expressed concern that they would be unable to find the app once we released it. By integrating the intervention into MyUFHealth, patients are able to log in to a system with which they are already familiar to access the app instead of downloading it from an unfamiliar Web-based source. Giving participants fewer tasks to complete before engaging with ALEX improves accessibility. Using MyUFHealth also allowed patients to view the intervention in the context of their relationship with their PCP and made requesting FIT easier as it could be done directly through MyUFHealth.

In addition, the app needed to be accessible to people who are hard of hearing and people with visual impairments. These requirements came out of the focus groups in Phase 1 and resulted in changes to the app’s interface. The computer science team prioritized easily-read subtitles so that participants could easily follow along with ALEX, and we selected the voices for ALEX in part based on focus group feedback as to which were the clearest and most easily understood.

### Conclusions

Ensuring that mHealth apps meet the needs of their target audience is an essential step toward widespread adoption. It is also a common shortcoming, with many mHealth apps being discarded by users shortly after initial usage owing to design failures that preclude their usefulness. Incorporating UCD principles into the design process of mHealth apps is one way to avoid this problem.

Our project used UCD principles in conjunction with expertise from communication science, computer science, clinical practitioners, and community members in an iterative process to create an mHealth app aimed at increasing CRC screening among adults aged 50 years and older. Through the phases of *needs investigation*, *prototype development*, and *evaluation*, we deliberately sought to highlight the opinions and concerns of community members as a way to increase the credibility, usability, effectiveness, and accessibility of the app. The overall product is one which aims to meet the needs of a variety of stakeholders as it moves through the clinical trial phase and into implementation across the health care system.

This study is not without limitations. A major limitation is lack of generalizability, with this project confined to 1 case study from the University of Florida. The iterative nature of UCD effected simultaneous collaboration among diverse academic disciplines, thereby presenting a potential challenge for replication in future research efforts where the culture and organizational structure may differ. However, stakeholder participation could be partially accomplished through centralized or remote participation, thus increasing the ability of other organizations that lack direct access to all key members to follow this blueprint.

Similarly, the study’s design by necessity incorporated the perspectives of the participants and researchers themselves. Although we made all efforts to remain reflexive, it is possible that an outside observer would have drawn different conclusions, presenting a possible threat to validity. In particular, the iterative nature of UCD means that assumptions are continually challenged and revised throughout the development process. This means the perspectives of team members evolved throughout the project as more information was uncovered and incorporated. This paper captures the end point of these evolutions, but it also means that the process may have looked different depending on when the participants were interviewed. We do not believe this represents a significant threat to the overall utility of the paper in describing the UCD process but individuals wishing to incorporate similar processes in their own work should be aware of and open to similar changes in their own understandings.

Similarly, the utility of mHealth apps is largely dependent on the surrounding medical environments and patient characteristics, which may vary by institution and population. From a structural perspective, for instance, involvement of health care providers might be necessary to provide trainings for patients with low technical literacy to ensure successful application of the app in the real medical settings, requiring additional staff and resources. From a patient characteristics perspective, characteristics such as age, health status, health literacy, and technological literacy may impact uptake of mHealth interventions. Although these characteristics are important for widespread dissemination and utilization of mHealth technology, they are beyond the scope of this study to explore. Regardless, the benefits of using mHealth to foster lifesaving preventative care outweigh such potential challenges, particularly when interventions incorporate UCD principles.

## Abbreviations

ALEX: Agent Leveraging Empathy for eXams
CRC: colorectal cancer
ECA: embodied conversational agent
FIT: fecal immunochemical testing
IT: information technology
mHealth: mobile health
PCP: primary care provider
UCD: user-centered design
UF: University of Florida
VHT: virtual human technology
